# COVID-19-Patientinnen und -Patienten in Deutschland: Expositionsrisiken und assoziierte Faktoren für Hospitalisierungen und schwere Krankheitsverläufe

**DOI:** 10.1007/s00103-021-03391-0

**Published:** 2021-07-29

**Authors:** Uwe Koppe, Hendrik Wilking, Thomas Harder, Walter Haas, Ute Rexroth, Osamah Hamouda

**Affiliations:** Abteilung für Infektionsepidemiologie, Robert Koch-Institut, Seestr. 10, 13353 Berlin, Deutschland

**Keywords:** COVID-19, SARS-CoV‑2, Assoziierte Faktoren, Hospitalisierung, Schwere Verläufe, COVID-19, SARS-CoV‑2, Associated factors, Hospitalizations, Severe disease

## Abstract

Das Severe Acute Respiratory Syndrome Coronavirus Type 2 (SARS-CoV-2) hat sich seit 2020 weltweit verbreitet. In Deutschland haben sich bis zum Ende Juni 2021 über 3,7 Mio. Menschen infiziert. Das Infektionsgeschehen betrifft jedoch nicht alle Bevölkerungsgruppen gleichmäßig. Einige Gruppen haben ein besonders hohes Risiko, sich zu infizieren oder nach der Infektion schwere Coronavirus-Disease-2019(COVID-19)-Verläufe zu erleiden.

Der vorliegende narrative Review vermittelt eine Übersicht über die Bevölkerungsgruppen in Deutschland, welche besonders von COVID-19 betroffen sind. Außerdem werden die bisher identifizierten Risikofaktoren beschrieben, die mit Krankenhausaufenthalten oder schweren COVID-19-Verläufen assoziiert sind.

SARS-CoV-2-Übertragungen finden an den verschiedensten Orten und in unterschiedlichen Situationen statt. Besonders betroffen erscheinen bestimmte berufliche Umgebungen, wie z. B. die Fleisch verarbeitende Industrie, aber auch Freizeitaktivitäten und Großveranstaltungen. Es wurden im Laufe der Pandemie Komorbiditäten identifiziert, die mit einem erhöhten Hospitalisierungsrisiko oder einem schweren COVID-19-Verlauf assoziiert sind, z. B. vorbestehende Lungen‑, Herz-Kreislauf- und Stoffwechselkrankheiten. Patientinnen und Patienten nach Organtransplantation und Personen mit Downsyndrom (Trisomie 21) haben nach einer SARS-CoV-2-Infektion das höchste Risiko für eine stationäre Behandlung.

Die identifizierten Rahmenbedingungen, die eine SARS-CoV-2-Verbreitung begünstigen, und das Wissen um besonders vulnerable Bevölkerungsgruppen bilden eine wichtige Evidenzgrundlage für die Planung von Präventionsstrategien und Maßnahmen zur Pandemiebekämpfung.

## Abstract (en)

The severe acute respiratory syndrome coronavirus type 2 (SARS-CoV-2) spread worldwide in 2020. By the end of June 2021, over 3.7 million people had been infected in Germany. The spread of the infection, however, is not evenly distributed across all parts of the population. Some groups are at a higher risk for SARS-CoV‑2 infections or severe coronavirus disease 2019 (COVID-19) trajectories than others.

This narrative review provides an overview of the parts of the population in Germany that are most affected by COVID-19. In addition, risk factors associated with hospitalization or severe courses of COVID-19 are identified.

SARS-CoV‑2 transmission may occur in various locations and settings. Professional settings, e.g., in the meat-processing industry, but also leisure activities and large public events are particularly affected. In the course of the pandemic, certain comorbidities associated with an increased risk for hospitalization or severe courses of COVID-19 have been identified. These include preexisting pulmonary, cardiovascular, and metabolic diseases. Patients with organ transplants and people with Down syndrome (trisomy 21) have the highest risk for hospitalization after SARS-CoV‑2 infection.

The identified settings that contribute to the spread of SARS-CoV‑2 and the knowledge about vulnerable groups with a higher risk for hospitalization or severe disease trajectories form an important evidence base for the planning of prevention strategies and the fight against the pandemic.

## Einleitung

Das Severe Acute Respiratory Syndrome Coronavirus Type 2 (SARS-CoV-2) hat seit Beginn der Coronavirus-Disease-2019(COVID-19)-Pandemie bis Ende Juni 2021 weltweit zu über 179 Mio. Erkrankungsfällen und über 3 Mio. Todesfällen geführt [1]. Dadurch, dass SARS-CoV‑2 leicht von Mensch zu Mensch übertragbar ist und zunächst in der Bevölkerung keine Immunität bestand, konnte sich das Virus rasch weltweit verbreiten. Für die Entwicklung geeigneter Präventions- und Interventionsstrategien ist es daher wichtig, die Risikofaktoren für eine SARS-CoV-2-Infektion und Charakteristika der COVID-19-Erkrankung zu verstehen.

Die Verbreitung des Virus in Deutschland verlief in mehreren Wellen und nicht alle Bevölkerungsgruppen waren zu jedem Zeitpunkt gleichmäßig betroffen. Zu Beginn der ersten Infektionswelle Anfang 2020 wurden SARS-CoV-2-Infektionen vor allem bei Reiserückkehrenden aus Italien, Österreich und von Kreuzfahrtschiffen festgestellt [2–4]. Im weiteren Verlauf kam es in weiteren Kontexten zu Ausbrüchen, wie etwa unter bestimmten Arbeitsbedingungen, bei Musikkonzerten oder Sportveranstaltungen [5–7]. Eine Vielzahl von Untersuchungen und Studien konnten bestimmte SARS-CoV-2-Infektionsrisiken identifizieren: Große Ausbrüche ereigneten sich z. B. an Orten, wo viele Menschen auf engem Raum zusammenleben, häufig assoziiert mit prekären Arbeits- und Lebensverhältnissen.

Das Lebensalter und andere Vulnerabilitäten der von COVID-19 betroffenen Bevölkerungsgruppen wirkten sich auch auf die Belastung des Gesundheitssystems hinsichtlich der Krankenhauseinweisungen aus. Während die erste Infektionswelle im Frühjahr 2020 in Deutschland mit den vorhandenen Ressourcen gut bewältigt werden konnte, waren die Krankenhäuser in anderen Ländern, beispielsweise Italien, deutlich überlastet [8]. Im weiteren Verlauf der Pandemie ab Ende 2020 wurden auch in Deutschland die Krankenhauskapazitäten sehr stark beansprucht [9].

Mit Voranschreiten der Pandemie und der Verfügbarkeit detaillierter medizinischer Daten gelingt es immer besser, assoziierte Faktoren zu identifizieren, die nach einer SARS-CoV-2-Infektion mit einem erhöhten Risiko für eine stationäre oder auch intensivmedizinische Behandlung einhergehen. Diese Informationen können dazu dienen, dass COVID-19-Patientinnen und -Patienten mit einem höheren Risiko für schwere Krankheitsverläufe frühzeitig identifiziert und engmaschiger medizinisch überwacht werden können. Gleichzeitig bilden diese Informationen im Rahmen der Impfstrategie auch eine Grundlage, um vulnerable Patientengruppen zu identifizieren und priorisieren.

Dieser Artikel bietet eine Übersicht über die Bevölkerungsgruppen in Deutschland, welche besonders von COVID-19 betroffen sind, und die bislang identifizierten Faktoren, die mit Krankenhausaufenthalten oder schweren COVID-19-Verläufen assoziiert sind.

## Expositionsrisiken und assoziierte Faktoren für SARS-CoV-2-Infektionen in Deutschland

Ein guter Kenntnisstand zu assoziierten Faktoren für eine SARS-CoV-2-Infektion ist notwendig für zielgerichtete nichtpharmazeutische antiepidemische Maßnahmen (z. B. bestimmte gesetzliche Vorschriften oder die AHA + L-Regel: Abstandhalten, Hygiene, Alltag mit Maske und Lüften), die das Auftreten schwerer Erkrankungen und damit die Überlastung der Ressourcen des Gesundheitssystems und Todesfälle verhindern sollen. Dies schließt Studien zu Orten und Umständen ein, an bzw. unter denen SARS-CoV‑2 übertragen wird. Eine besondere Herausforderung dabei ist, dass die Übertragungswahrscheinlichkeit nicht nur vom Ort und von Rahmenbedingungen, sondern auch von individuellen Umständen abhängt (Überdispersion, [10]). Darüber hinaus können Übertragungsereignisse in einigen Umgebungen (eher) mit Kontaktmustern und dem Ausmaß sozialer Netzwerke verbunden sein, die das Risiko einer Exposition und Weiterleitung erhöhen, als mit einem bestimmten Verhalten selbst. Es ist daher sehr schwierig bzw. kaum möglich, alle Umstände zu beschreiben, die einen Einfluss auf die Übertragung von SARS-CoV‑2 haben.

Grundsätzlich gibt es 2 unterschiedliche Hauptpfade der Übertragung:

- Aerosole (stark abhängig von Innenraum),
- Atemtröpfchen (stark abhängig von Entfernung).

Aerosole sind kleine Partikel, die beim Ausatmen abgegeben werden und sich im Raum verteilen können. Atemtröpfchen sind größere Partikel, die ebenfalls beim Ausatmen abgeben werden, aber schneller aus der Luft absinken als Aerosole. Diese Hauptpfade können unabhängig voneinander oder auch zusammen wirken, was die Differenzierung der jeweiligen Pfade oder die Nachvollziehbarkeit der Übertragungswege im Rahmen epidemiologischer Untersuchungen sehr schwierig macht. Die Übertragung über Oberflächenkontakt ist unabhängiger von Entfernung und Innenraum und erscheint weniger relevant. Andere Mechanismen, wie fäkal-orale oder Tier-Mensch-Übertragungen, erscheinen epidemiologisch unbedeutend.

Das Wissen zu den Einflussfaktoren einer SARS-CoV-2-Übertragung muss kontinuierlich erweitert werden. Die bisherige Evidenz zu Assoziation von Faktoren mit COVID-19-Erkrankungen stammt aus folgenden Quellen:

- Erfahrungen mit anderen respiratorischen Erregern und der biologischen Plausibilität,
- epidemiologische Assoziationen ermittelt in Ausbruchsuntersuchungen,
- Kontextfaktoren in Seroprävalenzstudien,
- Assoziationsstudien (analytisch-epidemiologische Studien).

Angesichts des pandemischen Auftretens von SARS-CoV‑2 konnte aus Public-Health-Sicht nicht auf Studienergebnisse mit hohen Evidenzgraden gewartet werden, bevor über Maßnahmen entschieden wurde. Daher basiert eine Vielzahl der initialen Empfehlungen in Bezug auf SARS-CoV‑2 auf der Kenntnis anderer respiratorischer Erreger (SARS-CoV‑1, Influenza) und der biologischen Plausibilität einer Übertragung von respiratorischen Erregern im Allgemeinen. Darüber hinaus wurde auch Evidenz aus technischen Versuchen, wie z. B. den Darstellungen zu Verbreitung von Atemtröpfchen und Aerosolen, herangezogen [11].

Zahlreiche Ausbruchsuntersuchungen zu COVID-19-Clustern konnten eine Vielzahl an epidemiologischen Bezügen identifizieren und damit Risikofaktoren charakterisieren. Eine Zusammenstellung findet sich in unterschiedlichen Veröffentlichungen [6, 7]. Darüber hinaus findet sich bei Buda et al. eine systematische Zusammenfassung des Infektionsumfelds von durch das Meldesystem erfassten COVID-19-Ausbrüchen in Deutschland [5].

International wurden Veranstaltungen wie große Musikkonzerte [12] und Sportereignisse, z. B. Profifußballspiele [13], als *Superspreading-Events* (Ereignisse, bei denen sich viele Personen anstecken) identifiziert. Das berufliche Umfeld von großen Ausbrüchen umfasst vorrangig den Niedriglohnsektor mit schwerer körperlicher Arbeit bei mangelnder Lüftung, wie z. B. die Fleisch verarbeitende Industrie [14], kann aber durchaus auch andere Berufsbereiche betreffen [15]. Auch im Umfeld von religiösen Veranstaltungen kam es häufig zu SARS-CoV-2-Infektionen, insbesondere bei hohen Teilnehmerzahlen und in beengten Räumen mit intensivem Kontakt. So standen z. B. mehr als 5000 COVID-19-Fälle mit einer Kirche in Südkorea im Zusammenhang, aber auch in Deutschland und anderen Ländern wurden ähnlich geartete Ausbrüche identifiziert [16, 17]. Ausbrüche mit hohen *Attack Rates* wurden ausgelöst, wenn in geschlossenen Räumen und in größeren Gruppen über längere Zeit gesungen wurde [18].

Ausbrüche mit dokumentierten Übertragungen im Außenbereich werden selten beschrieben. Die Übertragungswahrscheinlichkeit erscheint hier aufgrund der Luftbewegung und Verdünnungseffekte geringer, wenn der erforderliche Abstand eingehalten wird.

2020 standen die Inzidenz bei Kindern und das Vorkommen von Ausbrüchen in Schulen in einem engen Zusammenhang mit der Inzidenz in der Allgemeinbevölkerung [19, 20]. Mit der Verbreitung der leichter übertragbaren besorgniserregenden Virusvariante Alpha (B.1.1.7) ab Anfang 2021 erhöhten sich die Inzidenz bei Kindern im Kindergarten- und Schulalter und die Anzahl der Ausbrüche in Schulen und in der Kinderbetreuung. Trotz Unsicherheiten über die Stärke des Einflusses auf das Gesamtgeschehen zeigt sich, dass an Schulen und in der Kinderbetreuung – wie in allen gesellschaftlichen Bereichen mit einer Vielzahl an Kontakten – ein hohes Potenzial für SARS-CoV-2-Übertragungen besteht.

Eine große Limitation der Evidenz aus Ausbruchsuntersuchungen ist, dass in definierten Umgebungen, wie z. B. religiösen Veranstaltungen oder Arbeitsstätten, die epidemiologischen Links leichter identifiziert werden können als an Orten, bei denen sich untereinander unbekannte Menschen begegnen [21]. Zu Letzteren gehören z. B. großstädtische Shoppingmalls, der öffentliche Nahverkehr oder Großveranstaltungen. Darüber hinaus können Risikofaktoren für sporadische SARS-CoV-2-Infektionen außerhalb definierter Ausbrüche so nicht beschrieben werden. Eine weitere grundlegende Limitation sind fehlende Daten zur Umsetzung der Infektionsschutzmaßnahmen vor Ort, sodass ähnliche Situationen/Infektionsorte teilweise durchaus mit einem sehr unterschiedlichen Infektionsrisiko verbunden sein können. Beispielsweise fehlen Informationen zur Umsetzung der Schutzmaßnahmen in Schulen, die einen Vergleich der Häufigkeit von Schulausbrüchen in Abhängigkeit von guter bzw. fehlender/schlechter Umsetzung erlauben würden.

Seroprävalenzstudien können besonders stark betroffene Bevölkerungsgruppen identifizieren. Einen guten weltweiten Überblick über die Vielzahl von Studien bieten systematische Datensammlungen und Reviews [22–24]. Beispielhaft herausgehoben sei eine umfangreiche Studie aus den USA, die u. a. Essen in Innenräumen von Restaurants/Bars, Besuche von religiösen Einrichtungen, Fitnessstudios und bei haushaltsfremden Personen, Arbeiten in Innenräumen sowie Flugreisen während der Pandemie als assoziierte Faktoren für Seropositivität aufzeigen konnte [25]. Maskentragen war hier negativ mit dem Infektionsrisiko assoziiert, was die Schutzwirkung des Maskentragens unterstreicht.

Nur wenige Studien untersuchen, welchen Expositionsrisiken SARS-CoV-2-Infizierte vor ihrer Erkrankung im Vergleich zu Nichtinfizierten ausgesetzt waren. Solche Studien können gute Evidenz für relevante Fragestellungen liefern, sind aber aufgrund der oben genannten Probleme und unterschiedlichen Übertragungswege nicht unter kontrollierten Bedingungen durchführbar [26]. Die Übertragungsbedingungen von SARS-CoV‑2 können sich aus verschiedenen Faktoren und Bedingungen zusammensetzen und zeitlich wie lokal ändern, sodass sich nur sehr schwer kontrollierte Situationen definieren lassen, in denen Maßzahlen zur Assoziation gut erhoben werden können. Eine Fallkontrollstudie in England zeigte, dass Personen mit Tätigkeiten im Gesundheitswesen, in der Sozialfürsorge oder im Gastgewerbe eine erhöhte COVID-19-Erkrankungswahrscheinlichkeit hatten. Es gab zusätzlich auch Hinweise darauf, dass die Beschäftigung als Lagerarbeiter, in körpernahen Dienstleistungen, im Bausektor und im Bildungswesen mit einer erhöhten Wahrscheinlichkeit für eine Infektion verbunden war [27].

### Schlussfolgerung

Die bisherigen Erkenntnisse zu assoziierten Faktoren zeigen, dass die Übertragung von SARS-CoV‑2 an vielen Orten und unter verschiedensten Umständen erfolgen kann.

Innerhalb von epidemiologischen Studien sind Übertragungen in Haushaltsumgebungen sowie Pflege- und Gesundheitseinrichtungen gut untersuchbar und darüber hinaus sind größere Geschehen (*Superspreading-Events*) in öffentlichen Einrichtungen auffällig. Die Übertragungsrisiken von Fällen, die die Bindeglieder dieser Vorkommen darstellen (sporadische Fälle), verbleiben weitestgehend unklar. Hier wären die Anteile der SARS-CoV-2-Infektionen, die bestimmten Risiken zugeordnet werden können (attributables Risiko), in Zukunft von großer Bedeutung. Ein besseres Verständnis davon, wo eine Übertragung mit höherer Wahrscheinlichkeit stattfindet, ermöglicht verfeinerte und gezielte Maßnahmen bei der weltweiten Pandemiebekämpfung. Darüber hinaus ist auch der Einfluss von distalen Risikofaktoren, wie z. B. sozioökonomischen Ungleichheiten, für die Entwicklung von zukünftigen Public-Health-Konzepten sehr wichtig.

## Assoziierte Faktoren für eine Hospitalisierung bei COVID-19

Wie bereits erwähnt, untersuchten schon zu Beginn der Pandemie eine große Anzahl an Primärstudien, welche Vorerkrankungen bei COVID-19 mit einem erhöhten Hospitalisierungsrisiko einhergehen. Parallel hierzu wuchs aber auch die Anzahl der systematischen Übersichtsarbeiten (Reviews), die den Forschungsstand zu einer oder mehreren Vorerkrankungen zusammenfassen. In einer solchen Situation liegt es nahe, die Evidenzlage auf Basis der vorhandenen Reviews zusammenzufassen. Dieser als Umbrella-Review (oder *Overview of Reviews*) bezeichnete Ansatz hat seit einiger Zeit einen festen Platz im Methodenspektrum der evidenzbasierten Medizin.

Ein solcher Umbrella-Review zum Risiko, aufgrund einer Vorerkrankung wegen COVID-19 hospitalisiert zu werden, wurde zur Unterstützung der Entscheidungsfindung der Ständigen Impfkommission (STIKO) durchgeführt. Dazu wurde eine systematische Literaturrecherche in der COVID-19-Literaturdatenbank der Bibliothek des Robert Koch-Instituts (RKI) durchgeführt, die systematisch COVID-19-relevante Einträge in den Datenbanken PubMed und Embase (inkl. Medline) sowie auf den Preprint-Servern ArRvix, BioRvix, ChemRvix, MedRvix, Preprints.org, ResearchSquare und SSRN erfasst (letzte Suche: 11.12.2020). Ausgewertet wurden systematische Reviews in englischer oder deutscher Sprache (mit oder ohne Metaanalyse), die ab dem 01.01.2020 veröffentlicht worden waren [28]. Voraussetzung für den Einschluss war die Untersuchung mindestens einer Vorerkrankung und des Risikos für eine Hospitalisierung aufgrund von COVID-19. Aus diesen Arbeiten wurden die altersadjustierten Primärdaten der in Europa und Nordamerika durchgeführten Studien extrahiert. Dadurch, dass das Lebensalter der zentrale Risikofaktor für eine Hospitalisierung aufgrund von COVID-19 ist [29], können alle Studien mit Bezug auf andere Faktoren nur unter Berücksichtigung des Alters analysiert werden. Bei Vorliegen mehrerer Schätzer erfolgte eine Metaanalyse (Random-Effects-Modell). Für jede Beziehung bzw. jeden Endpunkt wurde die Vertrauenswürdigkeit der Evidenz mithilfe der Methodik der Grading of Recommendations Assessment, Development and Evaluation (GRADE) Working Group analysiert [30]. Verglichen wurde das COVID-19-bedingte Hospitalisierungsrisiko zwischen SARS-CoV-2-positiven Personen mit und ohne die jeweilige Vorerkrankung [28].

Insgesamt wurden 120 systematische Reviews identifiziert, aus denen Daten von 1134 Primärstudien analysiert wurden [28]. Von den 356 verbleibenden Studien wurden nach Durchsicht der Volltexte 102 europäische und US-amerikanische Studien in die Metaanalyse eingeschlossen. Die ersten Ergebnisse des Umbrella-Reviews wurden am 17.12.2020 in der STIKO-Empfehlung zur COVID-19-Impfung online vorab veröffentlicht [28].

Die wichtigsten Ergebnisse sind in Tab. 1 dargestellt. Es konnten Daten zum Hospitalisierungsrisiko für insgesamt 19 Grunderkrankungen bzw. Erkrankungsgruppen analysiert werden [28]. Zu jeder dieser Entitäten bzw. Patientengruppen waren jeweils mindestens eine bis maximal 10 Studien mit zwischen 500 und 22.725 Teilnehmenden verfügbar.

**Table 1:** 

Vorerkrankung	Effektschätzer (95 % KI)	I^2^ (%)	Studien (Anzahl, Quelle(n))	Gesamtzahl Patientinnen und Patienten	GRADE-Evidenzqualität
*Herz-Kreislauf-Erkrankungen*					
Arrhythmie oder Vorhofflimmern	1,41 (1,17–1,70)	0,00	3 [53–55]	13.544	+ + + +
Herzinsuffizienz	2,13 (1,24–3,67)	80,5	5 [53, 55–58]	19.995	+ + +
Koronare Herzkrankheit (KHK)	1,29 (1,11–1,51)	18,8	3 [53, 55, 58]	18.501	+ + + +
Arterielle Hypertonie	1,51 (1,27–1,81)	62,5	7 [53–59]	20.902	+ + +
*Metabolische Störungen*					
Diabetes mellitus	1,95 (1,72–2,20)	27,5	10 [53–62]	22.725	+ + + +
Adipositas (BMI > 30 kg/m^2^)	1,94 (1,73–2,18)	0,00	5 [53, 54, 56, 58, 63]	16.251	+ + + +
Chronische Nierenerkrankung	1,95 (1,31–2,91)	82,9	7 [53–55, 58–60, 63]	20.863	+ + +
Chronische Lebererkrankung	1,30 (1,10–1,60)	NA	1 [64]	500	+ + +
*Malignome*					
Krebserkrankung	1,18 (1,00–1,38)	14,0	6 [53–56, 60, 63]	16.051	+ + + +
*Lungenerkrankungen*					
Asthma bronchiale	1,32 (0,89–1,97)	57,1	4 [54–56, 63]	5766	+ +
Schwere chronische Lungenerkrankungen, z. B. COPD	1,76 (1,29–2,4)	7,9	5 [54–56, 59, 63]	6351	+ + + +
*ZNS-Erkrankungen*					
Zerebrovaskuläre Erkrankung oder Apoplex (Schlaganfall)	1,30 (1,03–1,64)	0,00	2 [53, 54]	9841	+ + + +
Demenz	1,31 (0,32–5,37)	93,2	3 [53–55]	13.544	+ +
Psychiatrische Erkrankungen (schwere Depression, Schizophrenie und bipolare Störung)	2,1 (1,2–3,7)	NA	1 [53]	11.122	+ + + +
*Erkrankungen des Immunsystems/iatrogene Immundefizienz*					
Autoimmunerkrankung	1,08 (1,01–1,17)	NA	1 [65]	9437	+ + +
Chronisch entzündliche Darmerkrankung	1,1 (0,8–1,51)	NA	1 [66]	464	+ + +
Rheumatologische Erkrankung	1,37 (1,08–1,73)	0	2 [53, 67]	9675	+ + + +
Zustand nach Organtransplantation	2,70 (1,30–5,40)	NA	1 [53]	9519	+ + +
Immundefizienz, Antikörpermangelsyndrome, IgA-Mangel	1,14 (0,75–1,75)	NA	1 [68]	1526	+ + +

*KI* Konfidenzintervall, *BMI *Body Mass Index, *COPD* chronisch obstruktive Lungenerkrankung, *ZNS* zentrales Nervensystem, *GRADE* Grading of Recommendations Assessment, Development and Evaluation, *I*^*2*^ Heterogenitätsmaß, *NA* nicht anwendbar

Bei den Herz-Kreislauf-Erkrankungen war nur die Herzinsuffizienz mit einem mehr als zweifach erhöhten Hospitalisierungsrisiko assoziiert, während die 3 übrigen analysierten Erkrankungen nur moderat erhöhte Risiken zeigten [28]. Patientinnen und Patienten mit Diabetes mellitus, Übergewicht oder chronischen Nierenerkrankungen hatten ein fast zweifach erhöhtes Risiko für eine Hospitalisierung aufgrund von COVID-19. Bezüglich chronischer Lebererkrankungen konnte nur eine vergleichsweise kleine Studie identifiziert werden, die ein leicht erhöhtes Risiko zeigte. Vorbestehende Krebserkrankungen erhöhten das COVID-19-bedingte Hospitalisierungsrisiko nur leicht, wobei der gemeinsame nach Studiengröße gewichtete Schätzwert aus 6 Studien statistisch nicht signifikant war. Auffällig war, dass Asthma bronchiale keinen signifikanten Risikofaktor darstellte, während das Risiko bei Patientinnen und Patienten mit chronisch obstruktiver Lungenerkrankung (COPD) deutlich erhöht war. Auffällig war auch das mehr als doppelt erhöhte Hospitalisierungsrisiko bei Patientinnen und Patienten mit psychischen Krankheiten. Bei den Erkrankungen des Immunsystems bzw. bei Immunsuppression konnte aus dem gesamten Datensatz die stärkste Risikoerhöhung erhoben werden für Patientinnen und Patienten mit Zustand nach Organtransplantation. Die Evidenzqualität nach GRADE war für alle untersuchten Zusammenhänge moderat (+ + +) bis hoch (+ + + +).

Keiner der untersuchten systematischen Reviews schloss Studien an Patientinnen und Patienten mit Downsyndrom (Trisomie 21) ein [28] obwohl bekannt ist, dass diese vermehrt schwere COVID-19-Verläufe zeigen [31–34]. Hierbei ist zu berücksichtigen, dass eine Trisomie 21 häufig mit Erkrankungen einhergeht, die als Risikofaktoren für einen schweren oder tödlichen COVID-19-Verlauf gelten, z. B. kongenitale Herzfehler oder Störungen des Immunsystems. So war in einer Studie das Risiko für COVID-19-bedingte Hospitalisierung bei Trisomie 21 fünffach erhöht [31].

Die teilweise fehlenden oder differierenden Definitionen der Komorbiditäten in den Studien sowie fehlende Angaben zu Therapie und Krankheitsstadium stellen Limitationen dieser Analyse dar [28]. Insbesondere die Verwendung von ICD-10-Codes (die solche Differenzierungen nicht vorsehen) erschwerte hier eine weitere Unterscheidung. Dort, wo es sich um Krankheiten mit heterogenem klinischen Bild handelt, könnte eine Unterschätzung des Einflusses schwerer Ausprägungen vorliegen. In den Studien wurden außerdem weder Interaktionen zwischen Alter und Vorerkrankung noch zwischen verschiedenen vorliegenden Vorerkrankungen untersucht. Eine weitere Einschränkung ergibt sich aus dem methodischen Vorgehen. Es wurden nur diejenigen Vorerkrankungen erfasst, die in den systematischen Reviews berichtet wurden, hier ergänzt um die Daten zur Trisomie 21. Andere, seltene Vorerkrankungen konnten hier nicht erfasst werden.

### Schlussfolgerung

Zusammengefasst zeigen diese Untersuchungen, dass das COVID-19-bedingte Hospitalisierungsrisiko nur bei einer vergleichsweise kleinen Anzahl von Vorerkrankungen deutlich, d. h. um das mindestens Zweifache, erhöht ist. Hierzu gehören insbesondere Patientinnen und Patienten nach Organtransplantation und Menschen mit Trisomie 21. Konsequenterweise sind diese Personengruppen im Rahmen der COVID-19-Impfempfehlung der STIKO in eine besonders hohe Prioritätsstufe aufgenommen worden (Tab. 1).

## Assoziierte Faktoren für schwere COVID-19-Verläufe

Die Definitionen für einen schweren COVID-19-Verlauf werden in der Literatur unterschiedlich festgelegt [35]. Ein häufig verwendeter Indikator für einen schweren COVID-19-Verlauf ist die Notwendigkeit einer intensivmedizinischen Behandlung. Man muss jedoch bedenken, dass die Kriterien für eine Aufnahme auf die Intensivstation nicht weltweit und nicht über den gesamten Pandemieverlauf vergleichbar sein müssen. Weitere häufig verwendete Definitionen für einen schweren COVID-19-Verlauf sind die Notwendigkeit einer invasiven Beatmung, das Vorliegen bestimmter medizinischer Parameter, wie z. B. eine eingeschränkte Sauerstoffsättigung des Blutes oder der Tod. Dadurch ergibt sich in der Literatur eine Vielzahl von Definitionen bezüglich der Definition schwerer COVID-19-Verläufe, wodurch die berichteten Effektschätzer für die jeweiligen Risikofaktoren stärker schwanken können. Daher beschränken wir uns in diesem Abschnitt auf die Ergebnisse aus einzelnen systematischen Reviews zu bestimmten assoziierten Faktoren, die die publizierten Primärstudien innerhalb eines bestimmten Zeitraums analysieren.

Der Anteil der hospitalisierten Patientinnen und Patienten mit schweren COVID-19-Verläufen schwankt zwischen den Studien nicht nur aufgrund unterschiedlicher Definitionen eines schweren Krankheitsverlaufs, sondern auch in Abhängigkeit der Charakteristika der untersuchten Patientinnen und Patienten, wie z. B. der Altersstruktur oder Komorbiditäten [35]. In Deutschland untersuchten mehrere Studien, wie häufig stationär aufgenommene COVID-19-Patientinnen und -Patienten schwere Krankheitsverläufe hatten, und verwendeten dazu Klinikdaten [36, 37] oder Abrechnungsdaten von Krankenkassen [38, 39]. In 2 Studien wurde die Notwendigkeit einer intensivmedizinischen Behandlung als Indikator für einen schweren COVID-19-Verlauf herangezogen. In beiden Studien lag der Anteil der hospitalisierten Patienten, bei denen dies erforderlich war, bei 21 % [36, 39]. Darüber hinaus benötigten zwischen 14 % und 17 % der hospitalisierten Patientinnen und Patienten eine invasive Beatmung [36, 38, 39] und zwischen 14 % und 26 % verstarben [36–39].

Das Risiko für einen schweren COVID-19-Verlauf ist jedoch nicht gleichmäßig über die Patientenpopulationen verteilt. Eine Metaanalyse von 59 Studien konnte zeigen, dass COVID-19-Patientinnen und -Patienten im Alter von über 70 Jahren ein höheres Risiko für intensivmedizinische Behandlung hatten als jüngere (relatives Risiko [RR] 2,70; 95 % Konfidenzintervall [95 % KI] 1,59–4,60) und auch ein höheres Mortalitätsrisiko (RR 3,61; 95 % KI 2,70–4,84; [40]). Darüber hinaus hatten Männer ein höheres Risiko für eine intensivmedizinische Behandlung als Frauen (RR 1,38; 95 % KI 1,09–1,74) und ebenfalls ein höheres Mortalitätsrisiko (RR 1,50; 95 % KI 1,18–1,91).

Bestimmte Symptome einer COVID-19-Erkrankung können einen Hinweis auf ein erhöhtes Risiko für schwere Krankheitsverläufe liefern. In einer Metaanalyse von 7 Studien war Atemnot mit einem deutlich erhöhten Risiko für eine intensivmedizinische Behandlung (Odds Ratio [OR] 6,55; 95 % KI 4,28–10,0) verbunden [41]. Auch Patientinnen und Patienten mit Diarrhö hatten ein erhöhtes Risiko für schwere COVID-19-Verläufe (OR 1,50; 95 % KI 1,10–2,03), die in dieser Analyse als Atemnot, Aufnahme auf die Intensivstation oder Tod definiert waren [42].

Da sich SARS-CoV‑2 in den Atemwegen vermehrt, ist das Vorliegen einer Lungenerkrankung ein naheliegendes Risiko für schwerere Krankheitsverläufe. In vielen Studien wurde gezeigt, dass eine COPD das Risiko für schwere Krankheitsverläufe erhöht: im Rahmen einer Metaanalyse von 29 Studien wurde geschätzt, dass das relative Risiko für Patientinnen und Patienten mit COPD 1,71-fach erhöht ist (95 % KI 1,49–1,97; [35]). Darüber hinaus hatten COVID-19-Patientinnen und -Patienten mit COPD auch ein 1,70-fach erhöhtes Risiko zu versterben. Auch Patientinnen und Patienten, die rauchen oder ehemals rauchten, hatten laut Metaanalyse ein erhöhtes Risiko für schwere Krankheitsverläufe verglichen mit Nichtrauchenden (RR 1,29; 95 % KI 1,18–1,42). COVID-19-Patientinnen und -Patienten mit einer Tuberkulose hatten ebenfalls ein erhöhtes Mortalitätsrisiko im Vergleich zu Erkrankten ohne Tuberkulose (RR 2,10; 95 % KI 1,75–2,51; [43]). Im Gegensatz dazu wurde für Personen mit Asthma bronchiale bislang nicht von einem konsistent erhöhten Risiko für schwere Krankheitsverläufe berichtet [44].

Personen mit Bluthochdruck hatten in einer Metaanalyse ein 1,46-fach erhöhtes Risiko für schwere Krankheitsverläufe (95 % KI 1,28–1,65) und ein 1,76-fach erhöhtes Risiko, zu versterben (95 % KI 1,58–1,96; [35]). Von besonderem Interesse ist in diesem Kontext das Protein Angiotensin Converting Enzyme‑2 (ACE-2), das eine Rolle bei der Regulation des Blutdrucks spielt und gleichzeitig auch der Eintrittsrezeptor für SARS-CoV‑2 in menschliche Zellen ist [45]. Zu Beginn der Pandemie war unklar, ob Personen, die antihypertensiv mit Inhibitoren des Angiotensinsystems (sog. ACE-Hemmer) behandelt werden, möglicherweise ein erhöhtes Risiko für schwere COVID-19-Verläufe haben. In aktuellen Studien wurde allerdings bislang kein erhöhtes Risiko bei Einnahme dieser Medikamente gefunden [46].

Kardiovaskuläre Vorerkrankungen erhöhen das Risiko für einen schweren Krankheitsverlauf (RR 1,54; 95 % KI 1,39–1,72) und die Mortalität (RR 2,08; 95 % KI 1,81–2,39). Darüber hinaus können im Verlauf der COVID-19-Erkrankung verschiedene kardiovaskuläre Komplikationen auftreten. In einer Metaanalyse konnte gezeigt werden, dass COVID-19-Erkrankte mit erhöhten Troponin-I-Werten (ein Indikator für eine Myokardschädigung) ein höheres Risiko für schwerere Krankheitsverläufe hatten (OR 5,22; 95 % KI 3,73–7,31; [47]). Auch Arrhythmien und thromboembolische Ereignisse wurden als Komplikationen beschrieben, die einen schweren oder auch tödlichen COVID-Verlauf verursachen können [48, 49]. Eine weitere Metaanalyse zeigte, dass die Konzentration der Gerinnungsparameter D-Dimere und Fibrinogen bei Patientinnen und Patienten mit schweren COVID-19-Verläufen erhöht war [50].

Das Vorliegen anderer Erkrankungen war ebenfalls mit einem Risiko für schwere COVID-19-Verläufe assoziiert, z. B. Diabetes mellitus (RR 1,46; 95 % KI 1,35–1,63) sowie chronische Nierenerkrankungen (RR 1,56; 95 % KI 1,31–1,86) und chronische Lebererkrankungen (RR 1,63, 95 % KI 1,23–2,15; [35]). Auch durch Adipositas (Body-Mass-Index ≥ 30) ist das Risiko für eine intensivmedizinische Behandlung erhöht [51].

### Schlussfolgerung

Systematische Reviews haben gezeigt, dass verschiedene Faktoren wie Alter, Geschlecht und Komorbiditäten mit schweren COVID-19-Verläufen assoziiert sind. Personen mit erhöhten Risiken sollten daher bevorzugten Zugang zu Impfungen und nichtpharmazeutischen Schutzmaßnahmen erhalten. Einschränkend bei den dargestellten Ergebnissen sind die unterschiedlichen Definitionen für einen schweren Krankheitsverlauf, wodurch die Vergleichbarkeit der Ergebnisse aus den jeweiligen Studien und die Genauigkeit der Maßzahlen für das Risiko nicht immer eindeutig feststellbar sind. Darüber hinaus können sich die berichteten Effektschätzer durch die Einbeziehung von Ergebnissen zukünftiger Studien verändern. Eine nicht im systematischen Review [35] berücksichtigte Primärstudie, die bei über 17 Mio. Personen Risikofaktoren für eine erhöhte Mortalität bei COVID-19 untersucht hat, kam zu vergleichbaren Ergebnissen [52]. In Zukunft wäre es hilfreich, wenn systematische Reviews durgeführt würden, die nur Studien mit bestimmten Kriterien für schwere COVID-19-Verläufe einbeziehen, sodass exaktere Effektstärken berechnet und Effekte besser abgeschätzt werden können.

## Fazit

Im Zuge der COVID-19-Pandemie wurden durch eine Reihe von Untersuchungen Rahmenbedingungen identifiziert, unter denen sich SARS-CoV‑2 gut ausbreiten kann. Diese bildeten die Basis für verschiedene Präventionsstrategien zur Begrenzung der weltweiten Verbreitung des Virus. Darüber hinaus konnten Bevölkerungsgruppen identifiziert werden, die für Krankenhauseinweisungen und schwere COVID-19-Verläufe besonders gefährdet sind. Das Wissen um diese vulnerablen Gruppen bildet eine wichtige Grundlage für die Planung antiepidemischer Maßnahmen.

## References

[1] WHO Coronavirus (COVID-19) dashboard. https://covid19.who.int/. Zugegriffen: 28. Juni 2021

[2] Yamagishi T, Kamiya H, Kakimoto K, Suzuki M, Wakita T (2020). Descriptive study of COVID-19 outbreak among passengers and crew on Diamond Princess cruise ship, Yokohama Port, Japan, 20 January to 9 February 2020. Euro Surveill.

[3] Perico N, Fagiuoli S, Di Marco F, Laghi A, Cosentini R, Rizzi M (2021). Bergamo and Covid-19: how the dark can turn to light. Front Med (Lausanne).

[4] Kreidl P, Schmid D, Maritschnik S, Richter L, Borena W, Genger JW (2020). Emergence of coronavirus disease 2019 (COVID-19) in Austria. Wien Klin Wochenschr.

[5] Buda S, an der Heiden M, Altmann D, Diercke M, Hamouda O, Rexroth U (2020). Infektionsumfeld von erfassten COVID-19-Ausbrüchen in Deutschland. Epidemiol Bull.

[6] Leclerc Q, Fuller N, Knight L, Funk S, Knight G (2020). What settings have been linked to SARS-CoV-2 transmission clusters? [version 2; peer review: 2 approved]. Wellcome Open Res.

[7] NERVTAG E (2020) SARS-COV‑2 trasnmission routes and environments. https://www.gov.uk/government/publications/sars-cov-2-transmission-routes-and-environments-22-october-2020. Zugegriffen: 21. Apr. 2021

[8] Remuzzi A, Remuzzi G (2020). COVID-19 and Italy: what next?. Lancet.

[9] DIVI Intensivregister https://www.intensivregister.de/#/index. Zugegriffen: 24. März 2021

[10] Endo A, Abbott S, Kucharski AJ, Funk S (2020). Estimating the overdispersion in COVID-19 transmission using outbreak sizes outside China. Wellcome Open Res.

[11] van Doremalen N, Bushmaker T, Morris DH, Holbrook MG, Gamble A, Williamson BN (2020). Aerosol and surface stability of SARS-CoV-2 as compared with SARS-CoV-1. N Engl J Med.

[12] Furuse Y, Sando E, Tsuchiya N, Miyahara R, Yasuda I, Ko YK (2020). Clusters of Coronavirus disease in communities, Japan, January-April 2020. Emerg Infect Dis.

[13] Corsini A, Bisciotti GN, Eirale C, Volpi P (2020). Football cannot restart soon during the COVID-19 emergency! A critical perspective from the Italian experience and a call for action. Br J Sports Med.

[14] Günther T, Czech-Sioli M, Indenbirken D, Robitaille A, Tenhaken P, Exner M (2020). SARS-CoV-2 outbreak investigation in a German meat processing plant. EMBO Mol Med.

[15] Böhmer MM, Buchholz U, Corman VM, Hoch M, Katz K, Marosevic DV (2020). Investigation of a COVID-19 outbreak in Germany resulting from a single travel-associated primary case: a case series. Lancet Infect Dis.

[16] James A, Eagle L, Phillips C, Hedges DS, Bodenhamer C, Brown R (2020). High COVID-19 attack rate among attendees at events at a church—Arkansas, March 2020. MMWR Morb Mortal Wkly Rep.

[17] Lee JY, Hong SW, Hyun M, Park JS, Lee JH, Suh YS (2020). Epidemiological and clinical characteristics of coronavirus disease 2019 in Daegu, South Korea. Int J Infect Dis.

[18] Hamner L, Dubbel P, Capron I, Ross A, Jordan A, Lee J (2020). High SARS-CoV-2 attack rate following exposure at a choir practice—Skagit County, Washington, March 2020. MMWR Morb Mortal Wkly Rep.

[19] Robert Koch Institut (2021) Täglicher Lagebericht des RKI zur Coronavirus-Krankheit-2019 (COVID-19) vom 20.04.2021. https://www.rki.de/DE/Content/InfAZ/N/Neuartiges_Coronavirus/Situationsberichte/Apr_2021/2021-04-20-depdf?__blob=publicationFile. Zugegriffen: 21. Apr. 2021

[20] Buchholz ULA, Otte im Kampe E, Lindahl M, Lewandowsky M, Hauer B, Pozo Martin F, El Bcheraoui C, Hanefeld J, Haas W (2021). Epidemiologie von COVID-19 im Schulsetting. Epidemiol Bull.

[21] Alpers K, Haller S, Buchholz U, RKI Feldteam (2021) Field investigations of SARS-CoV-2-outbreaks in Germany by the Robert Koch Institute, February-October 2020. Bundesgesundheitsblatt Gesundheitsforschung Gesundheitsschutz 64(4):446–453 10.1007/s00103-021-03296-y PMC7968857 33733292

[22] Arora RK, Joseph A, Van Wyk J, Rocco S, Atmaja A, May E et al (2020) SeroTracker: a global SARS-CoV‑2 seroprevalence dashboard. Lancet Infect Dis 21(4):e75–e76 10.1016/S1473-3099(20)30631-9 PMC7402646 32763195

[23] Galanis P, Vraka I, Fragkou D, Bilali A, Kaitelidou D (2021). Seroprevalence of SARS-CoV-2 antibodies and associated factors in healthcare workers: a systematic review and meta-analysis. J Hosp Infect.

[24] Rostami A, Sepidarkish M, Leeflang MMG, Riahi SM, Nourollahpour Shiadeh M, Esfandyari S et al (2020) SARS-CoV‑2 seroprevalence worldwide: a systematic review and meta-analysis. Clin Microbiol Infect 27(3):331–340 10.1016/j.cmi.2020.10.020 PMC7584920 33228974

[25] Nash D, Rane M, Chang M, Kulkarni SG, Zimba R, You W (2021). Recent SARS-CoV-2 seroconversion in a national, community-based prospective cohort of U.S. adults.

[26] Griffith GJ, Morris TT, Tudball MJ, Herbert A, Mancano G, Pike L (2020). Collider bias undermines our understanding of COVID-19 disease risk and severity. Nat Commun.

[27] Hiironen I, Saavedra-Campos M, Panitz J, Ma T, Nsonwu O, Charlett A (2020). Occupational exposures associated with being a COVID-19 case; evidence from three case-control studies.

[28] Vygen-Bonnet SKJ, Bogdan C, Harder T, Heininger U, Kling K, Littmann M, Meerpohl J, Meyer H, Mertens T, Schmid-Küpke N, Scholz S, Terhardt M, Treskova-Schwarzbach M, Überla K, van der Sande M, Wichmann O, Wicker S, Wiedermann U, Wild V, von Kries R (2021). Beschluss und Wissenschaftliche Begründung der Ständigen Impfkommission (STIKO) für die COVID-19-Impfempfehlung. Epidemiol Bull.

[29] Cohen JF, Korevaar DA, Matczak S, Chalumeau M, Allali S, Toubiana J (2020). COVID-19-related fatalities and intensive-care-unit admissions by age groups in Europe: a meta-analysis. Front Med (Lausanne).

[30] Harder T, Takla A, Eckmanns T, Ellis S, Forland F, James R (2017). PRECEPT: an evidence assessment framework for infectious disease epidemiology, prevention and control. Euro Surveill.

[31] Clift AK, Coupland CAC, Keogh RH, Hemingway H, Hippisley-Cox J (2021). COVID-19 mortality risk in down syndrome: results from a cohort study of 8 million adults. Ann Intern Med.

[32] Kantar A, Mazza A, Bonanomi E, Odoni M, Seminara M, Verde ID (2020). COVID-19 and children with Down syndrome: is there any real reason to worry? Two case reports with severe course. BMC Pediatr.

[33] Krishnan US, Krishnan SS, Jain S, Chavolla-Calderon MB, Lewis M, Chung WK (2020). SARS-CoV-2 infection in patients with down syndrome, congenital heart disease, and pulmonary hypertension: is down syndrome a risk factor?. J Pediatr.

[34] Malle L, Gao C, Hur C, Truong HQ, Bouvier NM, Percha B (2021). Individuals with Down syndrome hospitalized with COVID-19 have more severe disease. Genet Med.

[35] Dorjee K, Kim H, Bonomo E, Dolma R (2020). Prevalence and predictors of death and severe disease in patients hospitalized due to COVID-19: a comprehensive systematic review and meta-analysis of 77 studies and 38,000 patients. PLoS ONE.

[36] Nachtigall I, Lenga P, Jozwiak K, Thurmann P, Meier-Hellmann A, Kuhlen R (2020). Clinical course and factors associated with outcomes among 1904 patients hospitalized with COVID-19 in Germany: an observational study. Clin Microbiol Infect.

[37] Schiller M, Fisahn J, Huebner U, Hofmann P, Walther J, Riess S (2020). Coronavirus disease (COVID-19): observations and lessons from primary medical care at a German community hospital. J Community Hosp Intern Med Perspect.

[38] Karagiannidis C, Mostert C, Hentschker C, Voshaar T, Malzahn J, Schillinger G (2020). Case characteristics, resource use, and outcomes of 10 021 patients with COVID-19 admitted to 920 German hospitals: an observational study. Lancet Respir Med.

[39] Ludwig M, Jacob J, Basedow F, Andersohn F, Walker J (2021). Clinical outcomes and characteristics of patients hospitalized for Influenza or COVID-19 in Germany. Int J Infect Dis.

[40] Pijls BG, Jolani S, Atherley A, Derckx RT, Dijkstra JIR, Franssen GHL (2021). Demographic risk factors for COVID-19 infection, severity, ICU admission and death: a meta-analysis of 59 studies. BMJ Open.

[41] Jain V, Yuan JM (2020). Predictive symptoms and comorbidities for severe COVID-19 and intensive care unit admission: a systematic review and meta-analysis. Int J Public Health.

[42] Aziz M, Haghbin H, Lee-Smith W, Goyal H, Nawras A, Adler DG (2020). Gastrointestinal predictors of severe COVID-19: systematic review and meta-analysis. Ann Gastroenterol.

[43] Sarkar S, Khanna P, Singh AK (2021). Impact of COVID-19 in patients with concurrent co-infections: a systematic review and meta-analyses. J Med Virol.

[44] Skevaki C, Karsonova A, Karaulov A, Fomina D, Xie M, Chinthrajah S (2021). SARS-CoV-2 infection and COVID-19 in asthmatics: a complex relationship. Nat Rev Immunol.

[45] Tavares CAM, Bailey MA, Girardi ACC (2020). Biological context linking hypertension and higher risk for COVID-19 severity. Front Physiol.

[46] Bavishi C, Whelton PK, Mancia G, Corrao G, Messerli FH (2021). Renin-angiotensin-system inhibitors and all-cause mortality in patients with COVID-19: a systematic review and meta-analysis of observational studies. J Hypertens.

[47] Toraih EA, Elshazli RM, Hussein MH, Elgaml A, Amin M, El-Mowafy M (2020). Association of cardiac biomarkers and comorbidities with increased mortality, severity, and cardiac injury in COVID-19 patients: a meta-regression and decision tree analysis. J Med Virol.

[48] McBane RD, Torres Roldan VD, Niven AS, Pruthi RK, Franco PM, Linderbaum JA (2020). Anticoagulation in COVID-19: a systematic review, meta-analysis, and rapid guidance from mayo clinic. Mayo Clin Proc.

[49] Wen W, Zhang H, Zhou M, Cheng Y, Ye L, Chen J (2020). Arrhythmia in patients with severe coronavirus disease (COVID-19): a meta-analysis. Eur Rev Med Pharmacol Sci.

[50] Chaudhary R, Garg J, Houghton DE, Murad MH, Kondur A, Chaudhary R (2021). Thrombo-inflammatory biomarkers in COVID-19: systematic review and meta-analysis of 17,052 patients. Mayo Clin Proc Innov Qual Outcomes.

[51] Yang J, Ma Z, Lei Y (2020). A meta-analysis of the association between obesity and COVID-19. Epidemiol Infect.

[52] Williamson EJ, Walker AJ, Bhaskaran K, Bacon S, Bates C, Morton CE (2020). Factors associated with COVID-19-related death using OpenSAFELY. Nature.

[53] Reilev M, Kristensen KB, Pottegård A, Lund LC, Hallas J, Ernst MT (2020). Characteristics and predictors of hospitalization and death in the first 11 122 cases with a positive RT-PCR test for SARS-CoV-2 in Denmark: a nationwide cohort. Int J Epidemiol.

[54] Sisó-Almirall A, Kostov B, Mas-Heredia M, Vilanova-Rotllan S, Sequeira-Aymar E, Sans-Corrales M (2020). Prognostic factors in Spanish COVID-19 patients: a case series from Barcelona. PLoS ONE.

[55] van Gerwen M, Alsen M, Little C, Barlow J, Genden E, Naymagon L (2020). Risk factors and outcomes of COVID-19 in New York City; a retrospective cohort study. J Med Virol.

[56] Azar KMJ, Shen Z, Romanelli RJ, Lockhart SH, Smits K, Robinson S (2020). Disparities in outcomes among COVID-19 patients in a large health care system in California. Health Aff (Millwood).

[57] Ebinger JE, Achamallah N, Ji H, Claggett BL, Sun N, Botting P (2020). Pre-existing traits associated with Covid-19 illness severity. PLoS ONE.

[58] Petrilli CM, Jones SA, Yang J, Rajagopalan H, O’Donnell L, Chernyak Y (2020). Factors associated with hospital admission and critical illness among 5279 people with coronavirus disease 2019 in New York City: prospective cohort study. BMJ.

[59] Rentsch CT, Kidwai-Khan F, Tate JP, Park LS, King JT, Skanderson M (2020). Covid-19 testing, hospital admission, and intensive care among 2,026,227 United States veterans aged 54–75 years.

[60] Gu T, Mack JA, Salvatore M, Sankar SP, Valley TS, Singh K (2020). COVID-19 outcomes, risk factors and associations by race: a comprehensive analysis using electronic health records data in Michigan Medicine.

[61] Killerby M, Link-Gelles R, Haight S, Schrodt C, England L, Gomes D (2020). Characteristics associated with hospitalization among patients with COVID-19—Metropolitan Atlanta, Georgia, March–April 2020. MMWR Morb Mortal Wkly Rep.

[62] Mehra MR, Desai SS, Kuy S, Henry TD, Patel AN (2020). Cardiovascular disease, drug therapy, and mortality in Covid-19. N Engl J Med.

[63] Mendy A, Apewokin S, Wells A, Morrow A (2020). Factors associated with hospitalization and disease severity in a racially and ethnically diverse population of COVID-19 patients.

[64] Singh S, Khan A (2020). Clinical characteristics and outcomes of Coronavirus disease 2019 among patients with preexisting liver disease in the United States: a multicenter research network study. Gastroenterology.

[65] Burn E, Tebe C, Fernandez-Bertolin S, Aragon M, Recalde M, Roel E (2020). The natural history of symptomatic COVID-19 in Catalonia, Spain: a multi-state model including 109,367 outpatient diagnoses, 18,019 hospitalisations, and 5,585 COVID-19 deaths among 5,627,520 people.

[66] Singh S, Khan A, Chowdhry M, Bilal M, Kochhar GS, Clarke K (2020). Risk of severe Coronavirus disease 2019 in patients with inflammatory bowel disease in the United States: a multicenter research network study. Gastroenterology.

[67] D’Silva KM, Serling-Boyd N, Wallwork R, Hsu T, Fu X, Gravallese EM (2020). Clinical characteristics and outcomes of patients with coronavirus disease 2019 (COVID-19) and rheumatic disease: a comparative cohort study from a US‘hot spot‘. Ann Rheum Dis.

[68] Chhiba KD, Patel GB, Vu THT, Chen MM, Guo A, Kudlaty E (2020). Prevalence and characterization of asthma in hospitalized and nonhospitalized patients with COVID-19. J Allergy Clin Immunol.

